# Innate Immune Response against Hepatitis C Virus: Targets for Vaccine Adjuvants

**DOI:** 10.3390/vaccines8020313

**Published:** 2020-06-17

**Authors:** Daniel Sepulveda-Crespo, Salvador Resino, Isidoro Martinez

**Affiliations:** Unidad de Infección Viral e Inmunidad, Centro Nacional de Microbiología, Instituto de Salud Carlos III, 28220 Madrid, Spain; danisecre@hotmail.com

**Keywords:** HCV, vaccine, adjuvant, innate immune response, PRRs

## Abstract

Despite successful treatments, hepatitis C virus (HCV) infections continue to be a significant world health problem. High treatment costs, the high number of undiagnosed individuals, and the difficulty to access to treatment, particularly in marginalized susceptible populations, make it improbable to achieve the global control of the virus in the absence of an effective preventive vaccine. Current vaccine development is mostly focused on weakly immunogenic subunits, such as surface glycoproteins or non-structural proteins, in the case of HCV. Adjuvants are critical components of vaccine formulations that increase immunogenic performance. As we learn more information about how adjuvants work, it is becoming clear that proper stimulation of innate immunity is crucial to achieving a successful immunization. Several hepatic cell types participate in the early innate immune response and the subsequent inflammation and activation of the adaptive response, principally hepatocytes, and antigen-presenting cells (Kupffer cells, and dendritic cells). Innate pattern recognition receptors on these cells, mainly toll-like receptors, are targets for new promising adjuvants. Moreover, complex adjuvants that stimulate different components of the innate immunity are showing encouraging results and are being incorporated in current vaccines. Recent studies on HCV-vaccine adjuvants have shown that the induction of a strong T- and B-cell immune response might be enhanced by choosing the right adjuvant.

## 1. Introduction

### 1.1. Hepatitis C Virus

The hepatitis C virus (HCV) is a member of the *Hepacivirus* genus, *Flaviviridae* family. HCV is a virus with an envelope and a positive-sense single-stranded RNA genome. The HCV genome is translated into a large polyprotein that is processed in three structural (core, E1, E2) and seven non-structural (NS) mature proteins (p7, NS2, NS3, NS4A, NS4B, NS5A, and NS5B) [1]. HCV has a high genetic diversity that has given rise to seven major genotypes and more than 60 subtypes [2]. The level of genetic diversity is approximately 30% between genotypes and 15% between subtypes of the same genotype. HCV genotype 1 is the most prevalent, followed by genotypes 3, 4, and 2 [2]. Furthermore, HCV shows high genetic diversity in each of the individuals infected (up to 10%), since HCV exists as a viral quasispecies generated by the errors of the HCV polymerase and the HCV replication rate, which are very elevated [2,3,4]. HCV employs the generation of genetic variants, particularly viral quasispecies, to evade the adaptive immune response. Moreover, the adaptive immune response promotes the selection of variant viruses that escape T cell or antibody recognition, even though those variants may lose viral fitness [5].

### 1.2. Epidemiology of Hepatitis C

HCV is a bloodborne virus transmitted mostly through the sharing of injecting drug syringes and needles, incorrectly sterilized medical tools, transfusion of unsecured blood and blood products, and some sexual practices, mainly among men who have sex with men (MSM) [6]. After HCV inoculation and a variable incubation period, about 25% of people clear the virus spontaneously [7]. The vast majority of HCV-infected subjects pass the acute phase asymptomatically, and fulminant liver failure is rare (<1%). Chronic hepatitis C (CHC) is usually established in 75% of patients exposed to HCV, who remain positive for HCV RNA after the acute phase [7]. CHC progresses slowly over the years, during which liver fibrosis occurs, generating liver cirrhosis in approximately 10–20% of patients during 20–30 years of HCV infection. When cirrhosis is established, the infection can progress to end-stage liver disease and hepatocellular carcinoma [8].

HCV causes serious health problems that affect approximately 1% of the global population (71 million people are living with CHC), and about 1.75 million individuals have new infections each year [9,10]. Furthermore, in 2016 about 400,000 persons died from cirrhosis and hepatocellular carcinoma derived from hepatitis C infections [8,9].

### 1.3. Antiviral Treatments

The new direct-acting antivirals (DAAs) may cure more than 95% of people with HCV infection, which has dramatically changed the landscape of hepatitis C in the last years [6,11]. DAAs therapy is more effective, shorter, and safer than previous interferon (IFN) therapy [12]. This therapeutic advance could reduce the number of people infected with HCV and new HCV infections, allowing the eradication of hepatitis C [13]. This goal requires expanded HCV screening, unrestricted access to DAAs treatment, and risk behavior reduction, among other additional strategies. [13]. However, there are several limitations to this approach in the absence of an effective vaccine [14,15]. Thereby, between 2 and 5% of patients do not eliminate the HCV infection, and these patients can select resistant variants that limit the effectiveness of DAAs. Additionally, DAAs remain expensive and inaccessible to most developing countries, compromising the extensive use of this therapy. On the other hand, hepatitis C is asymptomatic in the vast majority of people, which means that around 80% of HCV-infected people are unaware of their infection. Besides, only 15% of those diagnosed are treated with antivirals [6]. Thus, all undiagnosed and untreated patients are potential transmitters of the virus and continue to develop the disease. Also, HCV infection is quite widespread in marginalized populations that do not usually access to the national health system, such as sex workers, MSM, people who inject drugs (PWIDs), and incarcerated people. Finally, successful DAAs therapy does not protect against HCV reinfection because the immunity generated during CHC is not usually protective [16].

### 1.4. Immune Response and Vaccines

HCV spontaneous elimination is associated with the induction of a strong and broad virus-specific cellular (CD4^+^ and CD8^+^ T-cells) and humoral (neutralizing antibodies) immune response, which is sustained beyond the HCV clearance [5]. Another common characteristic among people who eliminate the virus spontaneously is the presence of favorable genetic polymorphisms at *IFNL3-IFNL4* genes, such as rs12979860, rs8099917, and rs368234815 [5].

Vaccination of the population has been the only effective method to control the transmission of viral infections by providing collective immunity [17]. In the case of HCV, an effective vaccine would prevent reinfections after DAAs therapy. Besides, an adequate vaccine could help to eliminate the virus in the small percentage of patients who do not clear the HCV following DAAs treatment. Therefore, the development of a vaccine is likely to be necessary to control HCV infection worldwide. In this regard, it has been proposed that a vaccine that only reduces viral titers could be of great help to control the hepatitis C epidemic [18,19,20]. However, some “leaky vaccines” that do not entirely prevent viral transmission can select viral strains with enhanced pathogenicity [21]. This would create suitable conditions for the appearance of strains that cause more severe diseases in the unvaccinated. Therefore, the hepatitis C vaccine should ideally prevent transmission of HCV to block this type of evolution towards higher virulence [21].

Current approaches to hepatitis C vaccines are based on virus subunits (recombinant proteins, synthetic peptides, DNA, and viral vectors expressing various antigens), which are poorly immunogenic and should be administered in combination with potent adjuvants [22]. This review will summarize the current knowledge about vaccine adjuvants that activate the innate immune response, with a particular focus on the HCV vaccine development.

## 2. Hepatitis C Virus-Host Interaction: The Innate Immune Response

Several hepatic cells may sense HCV infection and contribute to the development of the antiviral immune response. These cells can be divided into two groups: (1) non-immune cells, which include hepatocytes, the main target for HCV replication; (2) professional hepatic immune cells, which include antigen-presenting cells (APCs), such as Kupffer cells (KCs) and dendritic cells (DCs), which link the innate immune response and the adaptive immune response. Most of these liver cells are also susceptible to adjuvant stimulation.

### 2.1. Hepatic Non-Immune Cells

#### 2.1.1. Hepatocytes

Hepatocytes constitute the primary target for HCV replication. Infected hepatocytes recognize viral single-stranded RNA (ssRNA) and double-stranded RNA (dsRNA) replication intermediates by the retinoic acid-inducible gene-I (RIG-I), toll-like receptor 3 (TLR3), and melanoma differentiation-associated gene 5 (MDA-5) [23,24]. Viral recognition activates an intracellular signaling cascade that ends in the phosphorylation of the IFN regulatory factor 3 (IRF3), regulatory factor 7 (IRF7), and nuclear factor kappa B (NF-κB), followed by the expression of antiviral and pro-inflammatory genes, mainly IFN type I (IFN-I; IFN-α and IFN-β) and type III (IFN-III; IFN-λ) [25,26,27]. 

#### 2.1.2. Cholangiocytes

Primary cholangiocytes are not susceptible to HCV infection. However, two cholangiocarcinoma cell lines have recently shown to maintain low HCV replication levels, indicating that cholangiocytes may constitute a new hepatic reservoir [28,29]. Cholangiocytes can produce inflammatory cytokines after stimulating several TLRs [30]. Therefore, cholangiocytes can respond to pathogen-associated molecular patterns (PAMPs) from HCV or danger-associated molecular patterns (DAMPs) released from HCV-infected hepatocytes.

#### 2.1.3. Hepatic Stellate Cells (HSCs)

Hepatic stellate cells (HSCs) do not support efficient HCV replication [31]. HSCs express most TLRs producing antiviral cytokines against HCV [32,33,34]. Thus, as with cholangiocytes, HSCs may respond to extracellular PAMPs or DAMPs. TLRs stimulation promotes HSCs differentiation to myofibroblast-like cells (MFLCs)-producing collagen, which drives liver fibrosis. HSCs store dietary retinoids and distribute them to other cells like hepatocytes. IFN stimulated genes (ISGs) expression in hepatocytes depends on retinoids supply, which is impaired after HSCs transformation in MFLCs. Furthermore, HSCs express transforming growth factor-beta (TGF-β) that may lead to a T-helper cell type 2 (Th2) immune response producing interleukin (IL)-10 and inducting a tolerogenic state in the liver [35].

#### 2.1.4. Liver Sinusoidal Endothelial Cells (LSECs)

HCV mediates a transient productive infection of liver sinusoidal endothelial cells (LSECs) that can sense HCV-PAMPs by RIG-I and TLRs, producing IFN-I and IFN-III [36,37]. These IFNs promote the release of exosomes that inhibit HCV replication in hepatocytes [36]. LSECs are considered an essential link between innate and adaptive immunity by secreting C-X-C motif chemokine 10 (CXCL10) that attracts activated T-cells to the liver [31,38]. LSECs can also act as APCs [38]. Finally, LSECs capture and transport HCV particles from sinusoid to the space of Disse and secrete bone morphogenetic protein 4 (BMP4), which facilitates HCV infection of hepatocytes [36,39,40,41].

### 2.2. Hepatic Professional Immune Cells

#### 2.2.1. Kupffer Cells (KCs)

KCs are resident liver macrophages that regulate tissue homeostasis, immune surveillance, and liver inflammation without being infected by HCV [42,43,44,45,46]. KCs phagocyte apoptotic bodies, DAMPs, or exosomes from infected cells. In the phagolysosomes, HCV PAMPs are sensed by TLR3/7/8/9. KCs can inhibit HCV replication producing pro-inflammatory cytokines, such as IL-1β, IL-6, IL-18, and tumor necrosis factor-alpha (TNF-α) [47,48,49,50,51,52,53], and secreting IFN-β through TLR3/4 signaling pathways [54]. In contrast, during chronic infection, KCs promote HCV infection by TNF-α-mediated up-regulation of the HCV receptors occluding and the cluster of differentiation 81 (CD81) [55]. KCs also promote a profibrotic function mediated by IL-1β and TNF-α [56,57]. Moreover, KCs can suppress antiviral functions of T-cells by producing galectin 9 (Gal-9), IL-10, programmed death-ligand (PD-L)-1, PD-L2, and TGF-β [46,49,58,59,60,61].

#### 2.2.2. Natural Killer (NK) Cells

NK cells are the earliest immune responders to HCV infection [62], producing IFN-γ, potently suppressing HCV replication, activating macrophages, and promoting T-helper cell type 1 (Th1) responses [63]. CD16^−^CD56^bright^ NK cells produce cytokines to recruit DCs and HCV-specific T-cells without cytolytic functions. CD16^−^CD56^bright^ cells cause inflammation, but not fibrosis [31,64]. CD16^+^CD56^dim^ NK cells are cytolytic (via perforin and granzyme), secrete low levels of cytokines, and induce fibrosis development [31,65]. Thus, both cytolytic and non-cytolytic functions of NK cells reduce the HCV viral load [66,67,68,69,70]. However, NK cells can also have a detrimental role against HCV infection by killing DCs and activated T-cells [71,72,73]. NKT cells are another group of NK cells that play a crucial role in the immune response against HCV by modifying the Th1/Th2 balance [74].

#### 2.2.3. Dendritic Cells (DCs)

DCs are a heterogeneous population that comprises classical or myeloid DCs (mDCs) and plasmacytoid DCs (pDCs). The primary role of mDCs is to present antigens to T-cells to induce immunity or tolerance [75,76], while pDCs produce high IFNs levels and other cytokines with immunostimulatory properties [77,78,79]. In the liver, DCs are susceptible to low levels of HCV infection [80]. DCs are activated through TLR3/7/8 recognition of HCV RNA to produce IL-12, IL-18, and IL-27, which support Th1 development and IFN-γ secretion [81]. On the other hand, HCV structural (core, and HCV envelope glycoprotein E1 and E2) and NS (i.e., NS3) proteins activate TLR2 on DCs, impairing its functionality [82,83,84].

## 3. Innate Immune Response and Vaccine Adjuvants

From a historical point of view, vaccines are the most cost-effective and successful measures against viral infections. Although live-attenuated vaccines are usually highly immunogenic, there are some concerns regarding their stability and safety. Therefore, modern vaccines focus on non-living antigens, such as recombinant proteins, peptides, or DNA. However, these vaccines are poorly immunogenic and need the co-administration of adjuvants to increase their efficacy. 

Adjuvants have been used in human vaccines for decades. However, the mechanisms of action of these classical general adjuvants remain incompletely understood [85]. Recent advances in the innate immunity knowledge and its link with adaptive immunity has allowed the development of new adjuvants that activate specific immunological pathways [86,87].

### 3.1. Innate Immunity Is Linked to Adaptive Immunity

**Figure 1:**
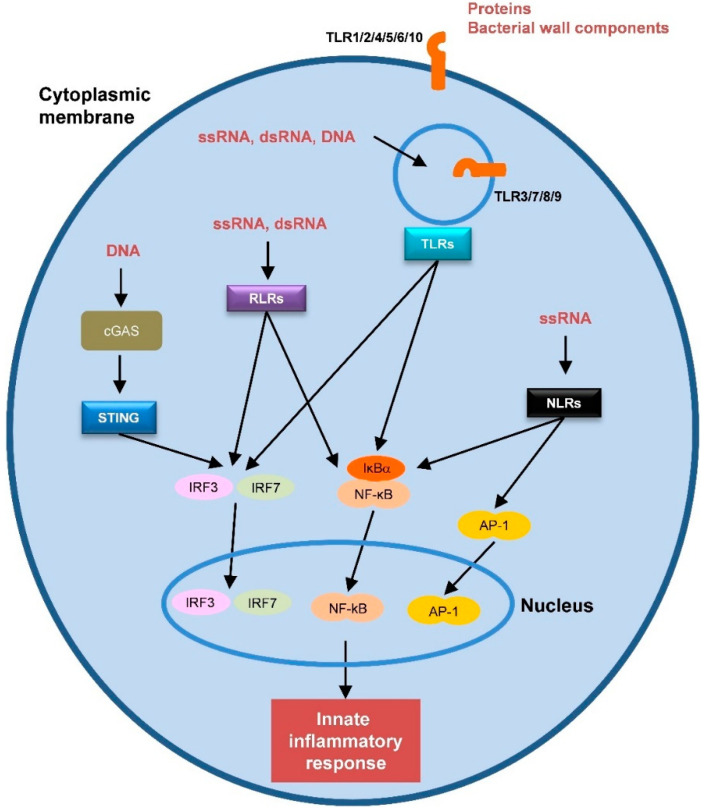
Schematic representation of the different pattern recognition receptors (PRRs) and their natural ligands. Transcription factors that are activated by the PRRs are also represented.

The innate immune response against viruses is initiated by the recognition of PAMPs (viral proteins and nucleic acids) by cellular pattern recognition receptors (PRRs) (Figure 1). DAMPs are molecules released by damaged or dying cells that also trigger the innate immune response. Activation of the innate response promotes adaptive immunity. APCs, particularly DCs, play a prominent role in this process [88]. DCs are well-equipped with several PRRs that recognize a variety of PAMPs, triggering intracellular pathways and producing pro-inflammatory cytokines and chemokines. Moreover, this recognition enhances the ability of DCs to present antigens and travel to lymphoid tissues where they interact with T- and B-lymphocytes to induce and modulate the adaptive immune response [89]. Therefore, PRRs stimulation by adjuvants could have a profound impact on vaccine performance [87,90,91].

### 3.2. Innate Immunity-Based Adjuvants

Several PRRs have been identified in humans, including TLRs, RIG-I-like receptors (RLRs), nucleotide-binding oligomerization domain (NOD)-like receptors (NLRs), C-type lectin receptors (CLRs) and cytosolic DNA sensors (CDs) (Figure 1) [88]. Most of the current innate immunity-based adjuvants target TLRs. In the following sections, we will describe some of the most relevant adjuvants targeting PRRs (Table 1).

#### 3.2.1. Toll-Like Receptors (TLRs)

There are ten functional TLRs in humans that can be classified into two groups: Plasma membrane receptors (TLR1, 2, 4, 5, 6, and 10) and endosome membrane receptors (TLR3, 7, 8, and 9) [92]. Both groups have an extracellular (plasma membrane receptors) or intra-vesicular (endosomal receptors) domain that mediates PAMPs recognition and a cytoplasmic domain that initiates downstream signaling (Figure 1). Endosomal receptors detect nucleic acids from pathogens inside the endosomes: TLR3 dsRNA, TLR7/8 ssRNA, and TLR9 unmethylated cytosine-phosphate-guanine (CpG) DNA. Surface TLRs sense diverse molecules of pathogens, including proteins and bacterial wall components [93] (Figure 1).

TLR3 is mostly expressed in mDCs and macrophages, detecting dsRNA and the dsRNA analog polyinosinic: polycytidylic acid (poly (I:C)) [94]. The use of poly (I:C) has been limited due to substantial side effects. Some of these side effects are reduced by stabilization with carboxymethylcellulose and poly-L-lysine while preserving positive effects on antibody production and CD4^+^ T-cell expansion [95,96,97].

The TLR4 agonist monophosphoryl lipid A (MPLA), a bacterial lipopolysaccharide (LPS) derivative, is a component of the current human papillomavirus (HPV) vaccine and a malaria vaccine candidate [98,99,100]. Glucopyranosyl lipid-stable emulsion (GLA-SE) is another TLR4 agonist that enhances T-cell response to an influenza vaccine [101] and is being tested in clinical trials in tuberculosis vaccines [102]. RC-529, an aminoalkyl glucosaminide 4-phosphate (AGP), is a synthetic monosaccharide mimetic of MPLA that improved the immunogenicity of the hepatitis B virus (HBV) vaccine Supervax [103]. Overall, TLR4 ligands promote Th1-based immune responses [104].

TLR5 ligands include bacterial flagellin, which has been used in influenza vaccines fused to the matrix protein or hemagglutinin. Fused proteins resulted in high antibody titers and protection, showing its great potential as an adjuvant in future vaccines [105,106]. 

TLR7 is expressed mainly in pDCs and B-cells, while TLR8 is expressed in many cell types [107]. Both TLR7 and TLR8 are activated by ssRNA [108,109]. Synthetic nucleoside analogs, such as imiquimod/R837 and resiquimod/R848, are recognized by TLR7 and TLR7/8, respectively [110,111,112]. Imiquimod/R837 has been administered as an adjuvant in a John Cunningham (JC)-virus vaccine to treat progressive multifocal leukoencephalopathy [113,114]. Imiquimod-treated patients developed a potent CD4^+^ T-cell response, cleared the virus, and showed substantial neurological recovery [113,114]. Imiquimod/R837 also increased the immunogenicity of an intradermal trivalent influenza vaccine [115]. Resiquimod/R848 has antiviral activity and induces potent Th1 and CD8^+^ T-cell responses [116,117]. However, the side effects of these adjuvants, such as fever and headache, are frequent, so improved formulations of TLR7/8 agonists are under development to reduce or eliminate them [118]. The 3M-052-SE, a less toxic modified imidazoquinoline TLR7/8 agonist, improves the breadth and functionality of HIV Env-specific antibody response in infant rhesus macaques compared to TLR4 agonists GLA-SE or alum [119].

TLR9 is highly expressed in pDCs and B-cells. TLR9 recognizes CpG dinucleotide motifs in unmethylated bacterial DNA [120]. Synthetic oligodeoxynucleotides (ODN) of about 20 bases with optimized CpG motifs (CpG-ODN) enhance antibody production and induce a polarized Th1 response, DC maturation, NK cells activation, and IFN-I production by pDCs [121,122,123,124,125]. Remarkably, CpG-ODN 1018 has been recently accepted by the Food and Drug Administration (FDA) as an adjuvant in a new HBV vaccine [126,127].

#### 3.2.2. Other Pattern Recognition Receptors (PRRs)

The cytosolic RLRs DExH/D-box RNA helicases RIG-I and MDA-5 recognize viral 5′-triphosphorylated RNAs and dsRNAs, respectively [128]. Similarly to TLR3, they also sense poly(I:C) and are potential targets for adjuvants.

Adjuvants stimulating NLRs, particularly nucleotide oligomerization domain 1 (Nod1) and 2 (Nod2), have been evaluated for a long time, and new ones are under development based on synthetic derivatives of peptidoglycan metabolites [129].

The stimulator of IFN genes (STING) recognizes the cyclic guanosine monophosphate-adenosine monophosphate dinucleotide (c-di-GAMP) to activate IRF3 and NF-κB transcription factors, which promotes DCs maturation and Th1 response [130,131]. While STING agonists as vaccine adjuvants have shown promising results in mice, the antiproliferative function of STING in human T-cells raises some concerns about its potential in human vaccines [132].

#### 3.2.3. Combined Adjuvant Formulations: Adjuvant Systems

Triggering several innate receptors seems to be more effective than activating a single one [133,134] (Table 2). Some of the most successful combinations of adjuvants are the so-called adjuvant systems AS01 (MPLA + QS-21 (a plant extract from *Quillaja saponaria* Molina, fraction 21) in liposomes), AS02 (MPLA + QS-21 in an oil-in-water emulsion), AS03 (squalene + α-tocopherol + polysorbate 80), and AS04 (MPLA + alum). Other interesting combinations of adjuvants are MF59 (squalene + polysorbate 80 + sorbitan trioleate) and AF03 (squalene + polyoxyethylene cetostearyl ether + mannitol + sorbitan oleate) [104].

AS01 has been incorporated in the malaria vaccine *Mosquirix* (sponsored and funded by GlaxoSmithKline, GSK) against *Plasmodium falciparum* [135,136]. AS01 is also included in the recently licensed vaccine *Shingrix* against herpes zoster (GSK) [137]. Several studies are currently testing the adjuvant efficacy of AS01 and AS02 in vaccines against HIV, tuberculosis, and HBV [104]. AS03 is used in pandemic hemagglutinin type 1 and neuraminidase type 1 (H1N1)/AS03 (*Pandemrix*, *Arepanrix*, GSK) and H5N1/AS03 (*Prepandrix*, GSK) influenza vaccines [138]. AS04 is in the *Fendrix* HBV vaccine (GSK) for individuals with chronic renal failure, and the *Cervarix* vaccine against HPV types 16/18 (GSK) [138]. The emulsions-based MF59 and AF03 are included in seasonal and pandemic influenza vaccines [104].

In summary, all these above-mentioned adjuvant combinations induce rapid activation of the innate immune response and increased antibody and T-cell responses [104,138].

## 4. Adjuvants in HCV Vaccines

Current HCV vaccines are based on different approaches that include proteins, peptides, DNA, virus-like particles and viral vectors [22,139]. The ideal vaccine should induce both a strong T- and B-cell immune response, but it is a challenging task. HCV vaccines focus primarily on NS proteins that predominantly induce T-cell immunity, and E1E2 proteins that primarily activate the B-cell response [139].

### 4.1. HCV Core and NS-Based Vaccines

Core and NS (NS3, NS4, and NS5) proteins are the most conserved proteins of HCV, the target of CD8^+^ T cells, and induce T cell-mediated immunity [140]. A strong and cross-reactive T-cell response is associated with the spontaneous resolution of acute HCV infection [141,142]. Moreover, studies in chimpanzees have shown that CD4^+^ and CD8^+^ T cells are essential in preventing HCV infection and reducing the chronicity [143,144,145,146,147].

A promising vaccine based on prime-boost protocols with chimpanzee adenovirus-3 and vaccinia Ankara virus, coding NS proteins (NS3-NS5B region), induced broad HCV-specific memory T-cells in human volunteers [148]. However, this vaccine showed no protection against the chronification of HCV infection in PWIDs in a phase 1/2 trial (clinicaltrials.gov identifier NCT01436357) [149], underlining the need for a vaccine stimulating both humoral and cell-mediated immunity.

A phase 1/2 trial of vaccination with autologous DCs pulsed with recombinant NS3 and core proteins combined with hIL-12 as an adjuvant has been completed for the treatment of patients with CHC (NCT03119025), and results are expected soon (Table 3). Recently, a phase 1 clinical trial of the DNA vaccine GLS-6150 (formerly VGX-6150) using *IFNL*3 gene as an adjuvant showed that the vaccine was safe and increased HCV-specific T-cell response in patients with CHC (NCT02027116) (Table 3). GLS-6150 consists of a mixture of DNA plasmids coding for the HCV NS3/NS4A, NS4B, and NS5A genes that demonstrated T-cell immunogenicity in C57BL/6 mice [150]. In the phase 1 trial, GLS-6150 immunization also reduced regulatory T (Treg) cell frequency [151]. This finding is relevant since Tregs are increased in patients with CHC, which may contribute to the attenuation of the HCV-specific T-cell response [152]. Furthermore, increased Tregs number persist after successful DAAs treatment [153]. Currently, a phase 1 trial with patients successfully treated with DAAs is ongoing (NCT03674125) to evaluate the safety and immunogenicity of the vaccine in this population (Table 3). Another phase 1 trial with a DNA vaccine encoding HCV NS3, NS4A, NS4B, and NS5A proteins (INO-8000) and hIL-12 as an adjuvant is underway to treat patients with CHC (NCT02772003) (Table 3).

Several recent preclinical studies with different HCV vaccine strategies accompanied by various adjuvants have generated promising results (Table 3). NS3, which has critical conserved immunodominant regions, is the most promising candidate for vaccination strategies in both DNA- and protein-based vaccines. Bacterial compounds are used as adjuvants to enhance the potency of DNA vaccines [154]. The DNA vaccine containing the HCV NS3 gene, with a truncated form of Listeriolysin O gene of *Listeria monocytogenes* as an adjuvant, induced significant cellular and humoral immune responses, lymphocyte proliferation and a Th1 profile in the BALB/c model [155]. 

**Table 3:** Adjuvants used in HCV core and non-structural protein-based vaccines in preclinical and clinical trials.

Adjuvant Name	Vaccine Type	Main Stimulated Immune Responses	Trial Stage	Ref
hIL-12	rNS3/core	N/A	Phase 1 completed (NCT03119025)	-
*IFNL3*	DNA plasmid GLS-6150 (NS3/NS4A, NS4B, NS5A)	T-cell response	C57BL/6 mice Phase 1 completed (NCT02027116)	[150,151]
*IFNL3*	DNA plasmid GLS-6150 (NS3/NS4A, NS4B, NS5A)	N/A	Phase 1 completed (NCT03674125)	-
hIL-12	DNA plasmid INO-8000 (NS3/NS4A, NS4B, NS5A)	N/A	Phase 1 ongoing (NCT02772003)	-
Truncated form of Listeriolysin O	DNA plasmid NS3	Ab response, T-cell response, Th1 type immunity	BALB/c mice	[155]
Hsp27, HR9 and Cady-2	DNA plasmid hsp27-NS3 + HR9/Protein rhsp27-rNS3 +Cady-2	Ab response, Th1 type immunity	BALB/c mice	[156]
Hp91 or hsp20	DNA plasmid NS3	Ab response, Th1 type immunity	BALB/c mice	[157]
Lenalidomide	DNA plasmid NS3	T-cell response, Innate response	BALB/c mice	[158]
Stork HBcAg	DNA plasmid NS3/4A	Memory T-cell response	C57BL/6J mice	[159]
PADRE, lipopeptide from *Neisseria meningiditis* and IL-2	rNS3, rNS4A/B, rNS5A	T-cell response, Th1 type immunity	BALB/c mice	[160]
G2 dendrimer	rNS3	Ab response, T-cell response	BALB/c mice	[161]
Multihydroxylated fullerene (C_60_(OH)_22_)	rNS3/core	Ab response, T-cell response	BALB/c mice	[162]
Sodium polyprenyl phosphate	rNS3, rNS5B	Ab response, Th1 type immunity	DBA/2J mice	[163]
CAF09	A mixture of 62 overlapping 20-mer peptides NS3	T-cell response	CB6F1 mice	[164]
CAF09	A mixture of six overlapping p7 peptides	Ab response, T-cell response	CB6F1, C57BL/6, and BALB/c mice	[165]
*Neisseria meningitidis* serogroup B outer membrane vesicles	rNS3/core	Ab response, Th1/Th2/Th17 type immunity	BALB/c mice	[166]
β-defensin	rNS3/4A, rNS5A, rNS5B	N/A	Immunoinformatics approaches	[167]

Ab: Antibody; CAF09: Cationic liposomes combined with the immunostimulants monomycolyl glycerol and poly(I:C); HBcAg: Hepatitis B core-related antigen; hIL-12: Human interleukin 12; Hp91: High mobility group box 1 protein (HMGB1) peptide; HR9: Histidine-rich R9; Hsp20: heat shock protein 20; Hsp27: Heat shock protein 27; *IFNL*3: Interferon lambda 3; N/A: Not available; PADRE: T-helper pan-human leukocyte antigen-DR isotype (HLA-DR)-binding epitope; Th1: T-helper cell type 1; Th17: T-helper cell type 17; Th2: T-helper cell type 2.

Priming with the DNA constructs encoding HCV NS3 and the heat shock protein 27 (hsp27) genes complexed with histidine-rich R9 (HR9) peptides followed by a boost with rhsp27-rNS3 proteins complexed with Cady-2 peptides elicited an enhanced Th1 cellular immune response with high antibody titers and an IFN-γ profile in BALB/c mice [156]. HR9 and Cady-2 are cell-penetrating peptides that, along with hsp27, were used as adjuvants to stabilize and facilitate gene delivery and to stimulate both innate and adaptive immunity. Similarly, a DNA vaccine containing the HCV NS3 gene, and using the high mobility group box 1 protein (HMGB1)-derived Hp91 peptide (Hp91) or hsp-20 as adjuvants, enhanced antibody titers, IFN-γ, and Th1 response compared to the commercially complete/incomplete Freund adjuvant (C/IFA) in a mouse model [157]. Hp91 and hsp-20 are endogenous molecules (DAMPs) that potentiate Th1 immunity. Another HCV DNA vaccine containing NS3 along with lenalidomide as an adjuvant increased cellular immunity, T-cell proliferation, and promoted innate immune response through NK activity and cytotoxic T-cell response in BALB/c mice [158]. Interestingly, this vaccine decreased the percentage of Treg cells. Lenalidomide is an immune-modulatory drug for the treatment of multiple myeloma with a substantial impact on the humoral and cellular immune system. Levander and colleagues designed an HCV DNA vaccine containing the NS3/4A gene and the avian HBV core gene-sequences (HBcAg) from stork as an adjuvant. The NS3/4A/stork-HBcAg vaccine enhanced HCV-specific IFN-γ and IL-2 and induced functional memory T-cell responses in transgenic and C57BL/6J mice [159].

The use of recombinant NS proteins as an HCV vaccine is an attractive approach since it allows a wide range of antigen design. Kuprianov et al. designed a recombinant HCV polyprotein vaccine consisting of cytotoxic T-lymphocyte epitopes of the NS3, NS4A/B, and NS5A proteins fused with Th pan HLA-DR-binding epitope (PADRE), the lipopeptide from *Neisseria meningiditis*, and IL-2 as adjuvants. This vaccine showed high immunogenicity, since it enhanced DCs stimulation and polarized them into a Th1 response, inducing IFN-γ and T-lymphocytes proliferation in BALB/c mice [160].

Nanotechnology has been introduced recently as dual adjuvant/delivery systems to potentiate both humoral and cellular responses. Nanoparticles have excellent biocompatibility, are easy to prepare, promote endocytosis, and improve immunogenicity and stability [168,169]. The rNS3 conjugated with a second-generation dendrimer as an adjuvant showed strong cellular responses, high antibody titers, and increased IFN-γ, IL-4, and cellular proliferation (stimulator of CD8^+^ T-cells) in BALB/c mice [161]. Remarkably, the immunogenicity effects of the dendrimer were higher than other commercially available adjuvants, such as human-compatible adjuvant Montanide ISA 720 (M720) and C/IFA. Another rNS3/core vaccine that contains multiple conserved CD4^+^ and CD8^+^ T-cell epitopes from HCV NS3 and core proteins were designed together with multihydroxylated fullerene (C_60_(OH)_22_) as adjuvant [162]. C_60_(OH)_22_ acts as an immune modulator and carrier that was successfully tested in a DNA HIV vaccine in mice [170]. The rNS3/core/C_60_(OH)_22_ vaccine was superior to the vaccine with alum adjuvant in a BALB/c mouse model because it promoted both specific humoral and cellular immune responses, induced IFN-γ and T-lymphocytes proliferation and activated CD4^+^ and CD8^+^ T-cells [162]. Masalova and colleagues designed and evaluated a vaccine with rNS3 and rNS5B of HCV as immunogens and sodium polyprenyl phosphate as an adjuvant in DBA/2J mice. Again, the adjuvant helped to exceed the immunostimulatory activity of alum adjuvant, increasing antibody and IFN-γ levels, and polarizing the immune response to Th1 type [163]. A vaccine, based on a mixture of 62 overlapping 20-mer peptides covering HCV NS3 and CAF09 adjuvant, induced polyfunctional T-cell responses in CB6F1 mice [164]. CAF09 consists of cationic liposomes combined with the immunostimulants monomycolyl glycerol and the TLR3 ligand poly (I:C) [171]. The CAF09 adjuvant was used by the same laboratory along with a mixture of six overlapping peptides from the HCV p7 protein, a crucial protein for efficient viral assembly and release [165]. This vaccine induced specific multifunctional cytokine-producing CD4^+^ and CD8^+^ T-cells and cleared hepatocytes expressing HCV p7 in different mice models.

Another interesting approach is the use of informatics tools to propose a multi-epitope vaccine with adequate adjuvants against HCV (Table 3). In this regard, truncated rNS3/core proteins were analyzed and evaluated using bioinformatic software [172]. The stability and antigenicity of these rNS3/core proteins, adjuvanted with *Neisseria meningitidis* serogroup B outer membrane vesicles (*NMB* OMVs), was evaluated in BALB/c mice [166]. This vaccine showed increased Th1/Th2/T-helper cell type 17 (Th17) immune responses (Th1 type more dominant than Th2 type) and high antibody titers compared to C/IFA and MF59 adjuvants. This confirms the validity of new integrated immunoinformatics tools to design safe and immunogenic multi-epitope vaccine-adjuvant combinations. Following this line, Ikram and colleagues proposed a multi-epitope vaccine with 16 conserved epitopes from the three NS proteins NS3/4A, NS5A, and NS5B and β-defensin as an adjuvant [167]. Molecular dynamic and molecular docking simulations suggest that the designed vaccine is stable and immunogenic with significant interactions with TLR3/8, although experimental validation is required. Finally, cholesterol modulation is recently emerging as a promising adjuvant strategy in CHC patients, since HCV clearance is enhanced by the immunoregulatory effects of cholesterol derivatives in the liver innate immune cells [173].

### 4.2. HCV E1E2-Based Vaccines

One of the most advanced HCV vaccines is based on recombinant E1E2 glycoproteins (rE1E2) derived from the genotype 1a HCV-1 isolate, adjuvanted with M59, which was administered to healthy volunteers in phase 1 human trial (NCT00500747) [174]. This vaccine was safe and induced a cross-reactive neutralizing antibody (nAb) response to several glycoprotein epitopes [174,175,176,177]. However, T-cell responses need to be improved by using better adjuvants. Furthermore, analysis of serum samples from human vaccinated volunteers, immunized rhesus macaques, and immunized C57BL/6J mice showed that antibody titers induced by rE1E2 are insufficient to neutralize diverse HCV isolates [178] (Table 4). Therefore, current clinical trials with promising results based on E1E2 glycoproteins are lacking.

However, many vaccine preclinical studies centered on E1, E2, or E1E2 proteins combined with different adjuvants are ongoing (Table 4). In this regard, rE1E2 immunogenicity has been tested in combination with varying adjuvants in mice [179,180]. The cyclic di-adenosine monophosphate (c-di-AMP) (a STING activator) or liposomes (archaeosomes) composed of sulfated lactosyl archaeol (SLA, a semi-synthetic archaeal lipid) induced a more potent Th1 type CD4^+^ T-cell response compared with M59 or alum/MPLA adjuvants [179]. Furthermore, c-di-AMP and archaeosomes elicited a strong nAb response, similar to those obtained with MF59 or alum/MPLA immunization [179]. Archaeosomes are phagocytosed by APCs, allowing antigen processing on the major histocompatibility complex I (MHC-I) pathway. At the same time, archaeosomes activate APCs and cytokine production [181,182], leading to a potent adjuvant activity [183]. Similar to MF59 and AS01 mimetics, SLA archaeosomes also induce long-lasting immunity in mice [180]. In addition to archaeosomes and c-di-AMP, enhanced CD4^+^ T-cells levels were observed in mice immunized with rE1E2 adjuvanted with an AS01 mimetic [180]. Further investigations on new combinations of SLA with other adjuvants, such as TLR agonists, will give us a more detailed picture of the adjuvant landscape.

**Table 4:** Adjuvants used in HCV E1E2-based vaccines in preclinical and clinical trials.

Adjuvant Name	Vaccine Type	Main Stimulated Immune Responses	Trial Stage	Ref
MF59	rE1E2	Ab response, CD4^+^ T-cell response	C57BL/6J mice Rhesus macaques Phase 1 completed	[174,175,176,177,178]
c-di-AMP STING or archaeosomes	rE1E2	Ab response, CD4^+^ T-cell response, Th1 type immunity	CB6F1 mice	[179]
SLA (Enc) or (Adm)-based archaeosomes	rE1E2	Ab response, CD4^+^ T-cell response, Th1 type immunity	C57Bl/6 x BALB/ c F1 mice	[180]
IMX313P	DNA plasmid expressing sE1/sE2	Ab response	BALB/c mice	[184]
R_4_Pam_2_Cys or E_8_Pam_2_Cys	Quadrivalent genotype VLP vaccine (E1, E2)	Ab response, T-cell response	BALB/c mice	[185]
ODN39M	Chimeric NS3EnvCo + rE2	Ab response, Th1 type immunity	BALB/c mice	[186]

Ab: Antibody; c-di-AMP: Cyclic di-adenosine monophosphate; E_8_Pam_2_Cys: Anionic TLR2 agonist-based lipopeptide (linear peptide containing glutamate residues plus S-[2,3-bis(palmitoyloxy)propyl] cysteine); IMX313P: Oligomerization domain from the chicken complement inhibitor C4b-binding protein; ODN39M: Oligodeoxynucleotide of 39 bases; R_4_Pam_2_Cys: Cationic TLR2 agonist-based lipopeptide (hyper-branched tetra arginine complex plus S-[2,3-bis(palmitoyloxy)propyl] cysteine); SLA: Sulfated lactosyl archaeol; SLA (Adm): E1E2 admixed with pre-formed empty archaeosomes; SLA (Enc): E1E2 encapsulated within SLA archaeosomes; STING: Stimulator of interferon genes; Th1: T-helper cell type 1; VLP: Virus-like particles.

A DNA vaccine encoding secreted HCV E1/E2 oligomers by fusion with the oligomerization domain of the chicken complement inhibitor C4b-binding protein (IMX313P) was designed and evaluated in BALB/c mice [184]. The sE1/sE2-IMX313P vaccine elicited an enhanced nAbs and cell-mediated responses against the envelope proteins when compared to separate (E1 or E2) immunogens. Oligomerization increases the structural stability of the proteins, but also may improve antigen uptake and favor prolonged antigen processing, all of which might enhance the immune response.

A quadrivalent genotype 1a/1b/2a/3a HCV virus-like particle (VLP) vaccine was designed and evaluated together with two TLR2 agonists, R_4_Pam_2_Cys (net positive charge) and E_8_Pam_2_Cys (negative charge) as adjuvants in BALB/c mice [185]. TLR2 agonists bind to protein antigens by electrostatic interactions enhancing antibody and T-cell responses. However, although total antibody titers were higher in mice inoculated with the E_8_Pam_2_Cys adjuvanted vaccine compared to VLPs alone, nAbs responses were not increased. 

An attractive vaccine based on a combination of a chimeric protein NS3EnvCo (containing selected conserved regions of HCV core, E1, E2, and NS3 proteins), a rE2 (E2.680) protein and ODN39M as an adjuvant has recently been designed [186]. NS3EnvCo/E2.680/ODN39M vaccine induced high antibody responses, cytotoxic T-lymphocyte response, IFN-γ, and Th1 type immunity in BALB/c mice.

In summary, the development of an HCV effective vaccine will require both a good immunogen and an appropriate adjuvant. To date, clinical trials on HCV vaccines are not entirely satisfactory, but the intense investigations to improve immunogens and adjuvant formulations are encouraging. For example, the increasing knowledge of E1E2 HCV glycoproteins’ structure and antigenic regions will undoubtedly help to find the proper vaccine. On the other hand, as described in the previous paragraphs, numerous adjuvants are being tested in preclinical studies, some of them with promising results. Still, the list of adjuvants tested in HCV vaccines is far from being complete and additional formulations can be tested in the light of its proven efficacy in vaccines against other pathogens (Table 1 and Table 2).

## 5. Conclusions

The development of new adjuvants is an exciting area of vaccinology guided by the increasing knowledge of how the innate immune response can be activated and how it triggers an adaptive immune response. There is also a growing need to find new and potent adjuvants due to the current tendency to generate vaccines based on poorly immunogenic subunits.

Global control of HCV will probably need a prophylactic vaccine inducing a strong T- and B-cell response. While significant efforts are being made in this field, results are encouraging but still not entirely successful. Recent studies strongly suggest that improved combinations of adjuvants may help to enhance HCV vaccine performance.

## Figures and Tables

**Table 1:** List of innate immunity-based adjuvants targeting pattern recognition receptors (PRRs).

PRR Type	Class of Activated Innate Receptor/Pathway	Adjuvant Name	Composition	Main Stimulated Immune Responses
TLRs	TLR3	Poly(I:C) or Poly(I:C) stabilized with poly-L-lysine	dsRNA analogs	Ab response, CD8^+^ T-cell response, Th1 type immunity
	TLR4	MPLA, GLA-SE, RC-529	MPL GLA AGP	Ab response CD8^+^ T-cell response Th1 type immunity
	TLR5	Flagellin fused to antigen	Bacterial flagellin	Ab response, Th1/Th2 response
	TLR7, 8 or both	Imiquimod/R837 (TLR7), Resiquimod/R848 (TLR7/8), 3M-052 (TLR7/8)	Imidazoquinoline analogs	Ab response, CD4^+^/CD8^+^ T-cell response, Th1 type immunity
	TLR9	CpG-ODN	Synthetic ODN with optimized CpG motifs	Ab response, CD8^+^ T-cell response, Th1 type immunity
NLRs	RIG-I MDA-5	M8, Defective interfering RNA	dsRNA analogs	Ab response, CD4^+^/CD8^+^ T-cell response
RLRs	Nod1 Nod2	iE-DAP, MDP	Bacterial peptidoglycan analogs	Ab response
CDs	STING	c-di-GAMP	Bacterial cyclic dinucleotides	Ab response, CD8^+^ T-cell response, Th1 type immunity

Ab: Antibody; AGP: Aminoalkyl glucosaminide 4-phosphate; c-di-GAMP: Cyclic guanosine monophosphate-adenosine monophosphate dinucleotide; CDs: Cytosolic DNA sensor ligands; CpG: Cytosine-phosphate-guanine; dsRNA: Double-stranded RNA; GLA: Glucopyranosyl lipid-adjuvant; iE-DAP: γ-D-glutamyl-meso-diaminopimelic acid; MDA-5: Melanoma differentiation-associated gene 5; MDP: Muramyl dipeptide; MPLA: Monophosphoryl lipid A adjuvant; NLRs: Nucleotide-binding oligomerization domain-like receptors; Nod1/2: Nucleotide oligomerization domain 1/domain 2; ODN: Oligodeoxynucleotide; Poly(I:C): Polyinosinic:polycytidylic acid; PRRs: Pattern recognition receptors; RIG-I: Retinoic acid-inducible gene-I; RLRs: RIG-I-like receptors; STING: Stimulator of interferon genes; Th1/Th2: T-helper cell type 1/type 2; TLRs: Toll-like receptors.

**Table 2:** List of selected current clinically tested combined adjuvant formulations.

Adjuvant Name	Composition	Vaccine Type	Main Stimulated Immune Responses
AS01	MPLA + QS-21 in liposomes	*Mosquirix*: Malaria, *Shingrix*: Herpes zoster	Ab response, CD8^+^ T-cell response, Th1 type immunity
AS02	MPLA + QS-21 in oil-in-water emulsions	HIV, tuberculosis, HBV	Ab response, Th1 type immunity
AS03	Squalene + α-tocopherol + polysorbate 80	*Pandemrix*, *Arepanrix*: Influenza H1N1, *Prepandrix*: Influenza pre-H5N1	Ab response, Th1/Th2 response
AS04	MPLA + aluminum hydroxide	*Fendrix*: HBV *Cervarix*: HPV 16/18	Ab response, Th1 type immunity
MF59	Squalene + polysorbate 80 + sorbitan trioleate	Influenza	Ab response, Th1/Th2 response
AF03	Squalene + polyoxyethylene cetostearyl ether + mannitol + sorbitan oleate	Influenza	Ab response

Ab: Antibody; H1N1: Hemagglutinin type 1 and neuraminidase type 1 (Influenza strain; aka swine flu); H5N1: Hemagglutinin type 5 and neuraminidase type 1 (Avian influenza A); HBV: Hepatitis B virus; HIV: Human immunodeficiency virus; HPV 16/18: Human papillomavirus type 16/18; MPLA: Monophosphoryl lipid A adjuvant; QS-21: A purified *Quillaja saponaria* Molina immunologic adjuvant fraction 21; Th1/Th2: T-helper cell type 1/type 2.
